# Refugee trauma measurement: a review of existing checklists

**DOI:** 10.1186/s40985-016-0024-5

**Published:** 2016-09-09

**Authors:** Erika Sigvardsdotter, Andreas Malm, Petter Tinghög, Marjan Vaez, Fredrik Saboonchi

**Affiliations:** 1 The Swedish Red Cross University College, Box 1059, 141 21 Huddinge, Stockholm Sweden; 2 Division of Insurance Medicine, Department of Clinical Neuroscience, Karolinska Institutet, Stockholm, Sweden; 3 Swedish Red Cross Treatment Center for Persons Affected by War and Torture, Malmö, Sweden

**Keywords:** Refugee trauma, Review, Trauma checklist, Review of trauma instruments

## Abstract

**Abstract:**

Studies have shown that a high proportion of refugees have been subjected to potentially traumatic experiences (PTEs). PTEs, including torture, are powerful predictors of mental ill health. This paper reports a review of refugee trauma history self-report measures used in population studies.

**Methods:**

A review of existing instruments and checklists, up to September 2015, was performed.

**Results:**

The types of measures for refugee trauma history vary from semi-structured interviews and medical records to extensive multi-item trauma-checklists. The Harvard Trauma Questionnaire (HTQ) was the most commonly used instrument for measuring trauma history among refugee populations. Few checklists included PTEs during the flight.

**Conclusion:**

Trauma history checklists are often used as a tool to control for background variables when studying refugees’ mental health and have mostly been developed in clinical or semi-clinical settings. There is a need for acceptable, reliable and valid brief checklists for measuring trauma in refugees, for the purpose of performing larger scale population studies.

## Background

A large share of refugees has been subjected to potentially traumatic experiences (PTEs), including torture. It is not uncommon that 20–40 % of non-clinical samples of refugee groups report having experienced torture [1]. PTEs and especially torture are powerful predictors of mental ill health, especially posttraumatic stress (PTS) symptoms, depression and anxiety [2, 3] and somatization [4]. Therefore, assessing trauma history is necessary when studying refugee health.

Reports of refugee trauma prevalence are generally found as part of a broader analysis of refugee mental health, where trauma is used as a background variable [1]. The majority of studies in this field are based on small convenience or consecutive samples, recruited in various community or clinical contexts. Steel et al. (2009) found that methodological factors such as smaller sample size, non-probabilistic samples and self-report measures have an impact on rates of generally yielded higher rates of posttraumatic stress disorder (PTSD) and depression [3]. While several explanations for this are possible, the same may be true for trauma history prevalence.

Measuring refugee trauma history in large-scale population studies requires consideration of typical trauma backgrounds and language- and culture-specific adaptation of items and instruments [5, 6], as well as adaption to the specific context, sample and type of study. Further, questions concerning traumatic events are sensitive requiring specific considerations to minimise the risk for retraumatisation, low response rates and non-response [7]. The choice of measure must be a result of considerations of the group studied and their background, context of data collection and type of sample.

### Purpose and aim

There is a need for studies of refugee trauma history prevalence based on larger random community samples, both in the context of refugee mental health studies and in its own right. The purpose of this study was to review and describe the existing trauma measures used with adult refugees in population studies.

## Method

The review of instruments was based on a systematic review of studies reporting prevalence rates of war-related PTEs in adult refugees in high-income countries. Studies published up to September 2015 were included. Psychiatric clinical populations were excluded. High-income countries were defined as the members of the Organisation for Economic Co-operation and Development (OECD). For the full details of the review method, see Sigvardsdotter et al. [1].

## Results

In the 42 articles reporting prevalence rates or torture and war-related PTEs in non-clinical settings, seven different instruments were used in order to measure trauma history. In addition, single trauma items, semi-structured interviews, medical records and sets of trauma items that were not properly described were used in a number of studies [1]. In the manual search of excluded articles, one additional relevant trauma instrument was found.

The eight trauma checklists that were found to have been used with adult refugees and where the developmental process is described in the literature are as follows: the Communal Traumatic Events Inventory (CTEI) [8]; the Comprehensive Trauma Inventory (CTI) [9]; the Harvard Trauma Questionnaire (HTQ), part 1 [10]; the Posttraumatic Stress Diagnostic Scale (PDS) part 1, [11]; the Stressful Life Events Screening Questionnaire (SLESQ) [12]; the Traumatic Life Events Questionnaire (TLEQ) [13]; the War Trauma Questionnaire (WTQ) [14] and the War Trauma Scale (WTS) [15]. An overview of the instruments can be found in Table 1.

**Table 1:** Existing refugee trauma measures described in the research literature

	No. of items	Target population	Development context	Conceptualisation of trauma	Inter rater reliability, test-retest reliability	Cronbach’s alpha	Type of measure
CTEI [8]	36	Bosnian refugees	Trauma literature		0.98, 0.93	0.9	Clinician administered
CTI [9]	104	Adult refugees	Empirically derived/community	“broad range of war-related events”/DSM IV	n/a, 0.83	0.99	Self-report
HTQ [10]	17	South East Asian refugees	Expert consensus/clinical context		0.93, 0.89	0.9	Self-report
PDS [11]	12	Western high-risk trauma populations	Empirically derived/community and clinical	Criterion A1, A2 of the DSM IV def of PTSD	n/a, n/a	0.85	Self-report
SLESQ [12]	13	General western population	Review of existing measures, community testing	Criterion A1 of the DSM IV def of PTSD	n/a, 0.89	n/a	Self-report
TLEQ [13]	16	General western population	Rational expert methods		n/a 0.74	n/a	Self-report
(C)WTQ [14]	28	Children affected by war in Lebanon	Trauma literature/Empirically derived	“outside the range of usual human experience”/DSM III	n/a, n/a	0.65	Self-report
WTS [15]	42	Cambodian adolescents refugees	Expert consensus/clinical context		n/a, n/a	n/a	Self-report

n/a, no data available

Three of these—CTEI, CTI, HTQ—were developed specifically in relation to adult refugee groups. The WTQ and WTS were initially developed in relation to childhood refugee trauma but have been used with adults [16, 17]. The remaining three, PDS, SLESQ and TLEQ, are instruments developed in relation to general (western) populations but have been used in refugee settings [18–21]. Several studies utilising these measures had modified them in some way, to better suit their study population or their study design or context.

Several of the included instruments are part of instruments measuring PTSD symptoms and report reliability or validity measures only for the measure as a whole, and no separate measure for the trauma check-list is available. Others do not report such measures at all.

### The CTEI

The CTEI is a 36-item, clinician-administered, clinically developed questionnaire designed specifically for the treatment of Bosnian refugees of ethnic cleansing. It is based on other screening instruments for refugees [22–24]. The CTEI has also been used with Kosovar refugees and was at that time shortened to 24 items after advice from caseworkers, to only include items likely to have occurred [25].

### The CTI

The CTI is a 104-item, self-report measure, developed to measure a broad range of war-related events in refugees. The 104 specific items are divided among 12 event-type scales, such as psychological and physical injury, detention and intentional abuse and deprivation and discrimination. Witnessing or hearing about traumatic events is inquired for as separate items. The response format allows respondents to check whether or not they experienced an event and, if so, how much impact the event had in terms of fear or threat. The CTI was developed by expert rational methods, in-depth interviews, and focus groups with Vietnamese and Kurdish refugees. [9]. The resulting 104-item checklist has shown acceptable temporal stability and internal consistency [26].

### The HTQ, part 1

The HTQ part 1 is a 17-item, self-report measure developed as a cross-culturally valid instrument to measure torture and trauma. The additional three parts of the instrument measures symptoms of posttraumatic stress disorder (PTSD). Examples of items are lack of food and water, loss of a loved one, rape, torture, brainwashing and an open catch-all item. The original response format allows the respondent to indicate whether the event happened to him/her, if he or she witnessed the event or heard about it, or none of these. The HTQ instrument is developed by expert consensus methods in a clinical psychiatric context with South East Asian refugees in the USA. It has shown excellent temporal stability and internal consistency [10].

More often than not, the instrument has been modified in different ways in studies of refugee trauma. In several cases, the reporting format has been modified, removing the options “witnessed” or “heard about” or both [27, 28]. Further, some studies have added questions on whether family members have experienced the asked for items [29, 30]. Items have been added [31–34], amended [27, 35] or removed [28], depending on the study population, research methods and context.

### PDS part 1

The PDS part 1 is a 12-item, self-report measure, developed to measure criterion A1 and A2 of the DSM IV definition of PTSD. The remaining three parts of the instrument measures symptoms of PTSD symptoms. PDS part 1 contains a checklist of 12 potentially traumatic events such as accidents and natural disaster, having experienced combat or war zone, sexual as well as non-sexual abuse, imprisonment and torture, including an “other” category, in which respondents are asked to indicate which of these events they have experienced or witnessed, and next, which of these has disturbed them the most in the past month. Criterion A2 of the DSM IV definition of trauma is assessed by four yes-no questions inquiring about physical injury to themselves or someone else and how the respondent felt at the time of the event (e.g. thinking that his/her life was in danger, thinking that someone else’s life was in danger, feeling helpless or terrified).

The trauma checklist is empirically developed through interviews with high-risk trauma populations in the USA such as PTSD-patients, residents of women’s shelters, rehabilitation residences, fire fighters, police and ambulance corps [11] and has later been used with refugee groups, on its own [18, 19] or in combination with other trauma measures [31].

### The SLESQ

The SLESQ is a 13-item, self-report measure developed as a general traumatic event screening questionnaire for use in non-treatment seeking samples in general western populations. It places less emphasis on disasters and more on traumata of an interpersonal nature and evaluates only the presence of PTEs, not the subjective criterion A2 of the DSM IV PTSD definition. Items include life-threatening illness and accident, robbery, traumatic bereavement, various kinds of sexual assault and physical abuse, and two catch-all items. It does not include items such as torture, prisoner of war, terrorist attack, accidents, or fires. The item pool was generated through a review of existing trauma checklists and pilot testing in community samples. Temporal stability and convergent validity were found to be adequate [12].

The SLESQ has been adapted for use used as a basis for developing a 14 Y/N questions to examine the traumatic incidents among Syrian refugees in Turkey [21].

### The TLEQ

The TLEQ is a 16-item (in a later version 23), self-report instrument for general trauma developed for use in primary care or emergency rooms in the USA. Examples of items are accidents, robbery, natural disaster, exposure to warfare, threat of death or serious bodily harm, childhood and intimate partner abuse, witness to family violence, and various types of sexual abuse. It includes one open “catch-all” item. The response format allows respondents to indicate for each item whether they experienced it “never”, “once”, “twice” or “more than twice” and “if more than twice, specify how many times”. Further, respondents are asked to specify whether they were injured and whether the experienced events evoked intense fear, helplessness or horror.

Items for TLEQ were generated through expert rational methods, and from the open-ended responses on the “other-trauma” item from more than 1000 completed versions of a preliminary checklist. Kubany et al. (2000) conducted additional studies in both community and clinical samples to test long- and short-term temporal stability (which were found to range between good, acceptable and problematic) and convergent validity, which was found to be good [13].

The TLEQ has been adapted for use with adult refugee groups, for example with Somali [20] and Bosnian [36] refugees in Canada and the USA to measure pre-migration trauma. In these studies, the checklist has been modified to better fit refugees’ experiences.

### The WTQ

The WTQ is a 28-item, self-report measure, initially developed as the CWTQ—the Childhood War-Trauma Questionnaire—and was distributed as a checklist for parents or legal guardians of children in Beirut to fill out. The final checklist of 28 items in nine categories such as exposure to shelling or combat, displacement, extreme poverty, physical injuries or handicap and witnessing violent acts. Each item had an open-ended response, where parents were asked to indicate how many times their child had experienced each event. The trauma conceptualisation was based on the DSM III definition of trauma in relation to PTSD. The checklist item pool was generated through a review of the literature, published life interviews with Lebanese children about their experiences during the war, and preliminary interviews with families with children, from various socioeconomic backgrounds [14].

The WTQ has been used with adult Kosovar Albanian refugees in the UK [16].

### The WTS

The WTS is a 42-item self-report measure, developed to be used with Cambodian adolescent refugees in the USA that had lived through the Pol Pot regime. The items are all worded in relation to the Pol Pot era (e.g. Were you ever tortured by the Khmer Rouge cadres or others?). The checklist was based on the researchers’ clinical experiences with the group. An interview version was checked against a self-report version showing moderate correspondence and good inter-rater reliability. The WTS was later used with adult Cambodian refugees [17].

## Discussion

This article reports a review of existing trauma measures used among adult refugees in non-clinical settings. Three (CTI, CTEI, HTQ) were developed specifically for adult refugee groups, of which two in clinical context. The HTQ part 1 was the most commonly used instrument to measure pre-migration PTEs in refugees [1] and has been described as a research standard in the field [37]. Three measures (PDS, SLESQ, TLEQ) were developed in relation to trauma in general western populations, of which all were developed wholly or partly in non-clinical contexts. Two (WTQ, WTS) were developed in relation to childhood refugee trauma, one in clinical context, but have been used with adult refugees.

Trauma checklists are often used as a tool to control for background variables when studying refugee health, mental health in particular. This is not surprising, given that trauma history is such an important factor in mental health, but has resulted in trauma checklists gaining less attention in their own right. Not seldom have the trauma checklists been developed in clinical settings rather than among community dwellers. This has influenced the way trauma is measured. Some of the more comprehensive trauma instruments have also attempted to measure the subjective responses to the mentioned events, corresponding to the criterion A2 of the DSM IV definition of PTSD. Such comprehensive lists must be used in a safe and trusting cooperation with respondents, in order to be able to care for any adverse reactions. In self-report measures where the contact between research-team and respondents is but brief, a shorter, less intrusive trauma checklist must be used.

Trauma checklists are often used to measure “frequency”, “amount” or “prevalence” of trauma in refugees. The result used in mental health analyses is usually the “number of trauma” calculated and reported as a mean and standard deviation. It must be remembered, however, that such a measure does not measure amount of trauma, but rather indicates a kind of variety of traumatic events, as it does not capture number of events in each category, length of incarceration or time spent in a war zone. Except for the checklists measuring the experienced levels of fear and horror, the severity of the event also cannot be captured.

The focus on mental health in measuring refugee trauma history means that other relevant factors are lost. For instance, in relation to health effects, the timing of an event is relevant, but rarely the context (before leaving home or during migration). That may, however, be relevant in other research contexts, especially in the current situation, where migration and refuge, in some cases, are becoming as dangerous and filled with horror as persecution or war.

### Limitations of the present report

The review of measures used with adult refugees in this study is focused on studies where prevalence rates of PTEs were reported. A wider selection may have found a greater number of trauma measures used with adult refugees.

## Conclusions

A review of trauma checklists used with refugees in population studies found that eight different instruments were used, of which the HTQ part 1 was the most common. Trauma checklists often gain less attention in their own right, as they often serve to measure background variables in mental health studies. Several of the most common measures are developed in clinical settings. In a situation where there is a great need for larger scale population studies concerning refugee trauma and mental health, there is a need for an acceptable, reliable and valid brief measure for refugee trauma history.

## Abbreviations

CTEI: Communal Traumatic Events Inventory
CTI: Comprehensive Trauma Inventory
DSM: Diagnostic and Statistical Manual of Mental Disorders
HTQ: Harvard Trauma Questionnaire
OECD: Organisation for Economic Co-operation and Development
PDS: Posttraumatic Stress Diagnostic Scale
PTE: Potentially traumatic event
PTSD: Posttraumatic stress disorder
SLESQ: Stressful Life Events Screening Questionnaire
TLEQ: Traumatic Life Events Questionnaire
WTQ: War Trauma Questionnaire
WTS: War Trauma Scale
